# A clinicopathological analysis of 153 acral melanomas and the relevance of mechanical stress

**DOI:** 10.1038/s41598-017-05809-9

**Published:** 2017-07-17

**Authors:** Yi-Shuan Sheen, Yi-Hua Liao, Ming-Hsien Lin, Jau-Shiuh Chen, Jau-Yu Liau, Yu-Ju Tseng, Chih-Hung Lee, Yih-Leong Chang, Chia-Yu Chu

**Affiliations:** 1 Graduate Institute of Pathology, College of Medicine, National Taiwan University and Department of Pathology, National Taiwan University Hospital, 7 Chun-Shan South Road, Taipei, 10002 Taiwan; 2 Department of Dermatology, National Taiwan University Hospital and National Taiwan University College of Medicine, 7 Chun-Shan South Road, Taipei, 10002 Taiwan; 3 Graduate Institute of Clinical Medicine, College of Medicine, National Taiwan University, 7 Chun-Shan South Road, Taipei, 10002 Taiwan; 4 Department of Surgery, National Taiwan University Hospital Hsin-Chu Branch, 25 Lane 442, Sec. 1, Jingguo Road, Hsin-chu, 30059 Taiwan; 5 Department of Dermatology, Kaohsiung Chang Gung Memorial Hospital and Chang Gung University College of Medicine, 123 Da-Pi Road, Kaohsiung, 83301 Taiwan

## Abstract

The pathogenesis of melanomas emerging in plantar surfaces remains unclear; however, mechanical stress has been reported to increase the formation of melanomas. In this study, we conducted a multicenter retrospective analysis of 153 acral melanomas diagnosed between 2000 and 2015 in Taiwan. The male-to-female ratio of the patients in question was 1:1.28, and the mean age at diagnosis was 68 years. We examined whether melanomas which developed in different areas of the patients’ soles differed in their associations with various clinicopathological characteristics and survival. Testing by goodness of fit indicated that stress-bearing areas were significantly more conducive to the generation of melanomas than non-stress-bearing areas (*P* < 0.0001). More specifically, compared to the arch, the rear of the foot and front of the foot were significantly more conducive to the generation of melanomas (*P* < 0.0001 and *P* < 0.0001, respectively). The distribution pattern was not associated with differences in age, gender, right/left foot involvement, ulceration, mitosis, lymph node metastasis, tumor thickness, or stage. The overall, distant metastasis-free, and recurrence-free survival rates did not differ significantly between the stress-bearing and non-stress-bearing areas. Furthermore, while acral melanomas tended to develop on stress-bearing areas, the distribution pattern was not associated with the prognostic index or survival.

## Introduction

In Asia, over half of all melanomas are acral melanomas^1^. Melanomas on sun-exposed areas are seen less frequently in Asians than in Caucasians^2,3]^. Relatedly, ultraviolet radiation does not appear to be associated with melanomas in Asian populations^4^. Other typical risk factors for melanoma, such as a personal or family history of previous melanoma, fair skin, and preexisting melanocytic nevi, seem to be less applicable in acral melanomas^5^. Meanwhile, the relevance of mechanical stress to acral melanomas has previously been reported with respect to Scots, Japanese, and Koreans patients^5–7^. To our knowledge, however, no studies regarding the relationship between mechanical stress and acral melanomas in Taiwanese patients over a given time period have previously been reported. Therefore, we established a clinical database of acral melanomas in Taiwanese patients and analyzed whether any differences in frequency and clinicopathological characteristics existed among melanomas that developed in different areas of the sole. In this study, the associations between the distribution pattern of the melanomas and the overall survival (OS), distant metastasis-free survival (DMFS), and recurrence-free survival (RFS) rates were evaluated^8–11^.

## Results

Summarized data regarding 153 melanoma patients are listed in Supplementary Table S1. The cohort included 67 (43.8%) men and 86 (56.2%) women with a mean age of 68 years (median: 69 years; range: 26–92 years). Most of the patients (88.2%) were diagnosed after their sixth decade of life. The melanomas which developed on volar surfaces were observed in the following frequencies: 83.0% were located on soles and 17.0% were located on toes. The plantar lesions were distributed almost equally between the right and the left side. Ulceration was present in 32.7% (50/153) of the patients. The Breslow thickness of the lesions was *in situ* in 21 cases, 1.00 mm or less in 23 cases, 1.01 to 2.00 mm in 37 cases, 2.01 to 4.00 mm in 33 cases, and 4.01 mm or more in 39 cases. The mean Breslow thickness of invasive melanoma was 4.1 mm. At presentation, 13.1% of the cases presented melanoma *in situ*, 68.6% of the cases were at stage I or II, and 18.3% of the cases were at stage III or IV. After diagnosis, the mean duration of patient follow-up was 4.1 years.

**Figure 1:**
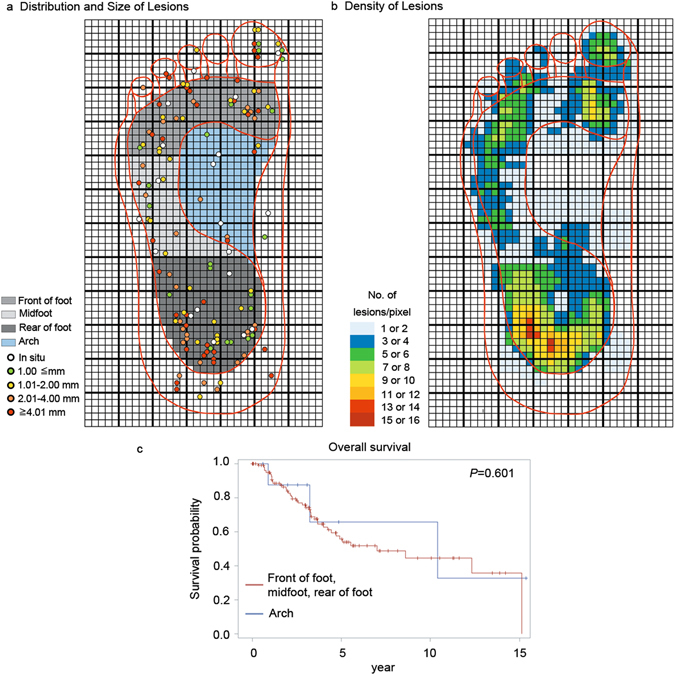
Distribution of acral melanomas in the plantar area. (**a**) The four areas of the sole are illustrated. The center of each lesion is marked and color-coded conforming to its Breslow thickness. (**b**) The number of lesion areas in each pixel is demonstrated on a color scale. Each pixel measures 5 mm by 5 mm. (**c**) Kaplan-Meier curves of survival in 114 acral melanomas. No overall survival differences were found between melanomas developed on stress-bearing and non-stress-bearing areas (*P* = 0.6, log-rank test).

According to the process used in a previous study, the width of the foot was adapted in each clinical figure to 26 cm through digital magnification and then the center and margin of each lesion were schemed on grids partitioned into 0.25-cm^2^ pixels (Fig. 1a and b)^6^. We also divided the sole into 5 regions (front of the foot, midfoot, rear of the foot, arch, and borders) based on the same previous study^6^. For the 114 lesions located on the soles of the patients (excluding those on the borders), the locations of the melanomas were as follows: 52 lesions (0.9 lesions per square centimeter) were located on the rear of the foot, 37 (0.82 per square centimeter) were located on the front of the foot, 16 (0.46 per square centimeter) were located on the midfoot, and 9 (0.21 per square centimeter) were located on the arch. The density of tumors on the entire plantar surface was 0.46 lesions per square centimeter. The front of the foot, midfoot, and rear of the foot are considered to be stress-bearing regions, while the arch is regarded as the non-stress-bearing portion. Testing by goodness of fit indicated that the stress-bearing areas were significantly more conducive to the growth of melanomas than non-stress-bearing areas (*P* < 0.0001) (Supplementary Fig. S1). More specially, compared to the arch, the rear of the foot and front of the foot were significantly more conductive to the generation of melanomas (*P* < 0.0001 and *P* < 0.0001, respectively), but the midfoot was not significantly more conductive to the generation of melanomas (*P* = 0.059). Moreover, the distribution pattern of the melanomas was not associated with age, gender, right/left side, ulcer, mitosis, lymph node metastasis, tumor thickness, or stage (Table 1).

**Table 1:** Factors associated with the stress- and non-stress-bearing areas.

Parameters	Stress-bearing area (n = 105)	Non-stress-bearing area (n = 9)	*P* value
Gender			1.00^†^
Male	50 (47.6%)	4 (44.4%)	
Female	55 (52.4%)	5 (55.6%)	
Age, y^*^	69.03 ± 13.82	66 ± 8.19	0.52
Location			0.73^†^
Left	57 (54.3%)	4 (44.4%)	
Right	48 (45.7%)	5 (55.6%)	
Thickness (n = 99)^*‡^	3.91 ± 4.05	5.53 ± 3.05	0.34
*In situ*	12 (11.4%)	3 (33.3%)	0.31^†^
≤1	15 (14.3%)	0 (0.0%)	
1.01–2.0	27 (25.7%)	1 (11.1%)	
2.01–4.0	25 (23.8%)	2 (22.2%)	
>4	26 (24.8%)	3 (33.3%)	
Ulceration			1.00^†^
Present	33 (31.4%)	3 (33.3%)	
Absent	72 (68.6%)	6 (66.7%)	
Mitosis (n = 44)^‡^			0.76^†^
<1	14 (34.1%)	1 (33.3%)	
1–6	20 (48.8%)	1 (33.3%)	
>6	7 (17.1%)	1 (33.3%)	
Lymph node metastasis			0.67^†^
Present	19 (18.1%)	2 (22.2%)	
Absent	86 (81.9%)	7 (77.8%)	
AJCC stage			0.24^†^
0	11 (10.5%)	3 (33.3%)	
I	32 (30.5%)	1 (11.1%)	
II	43 (41%)	3 (33.3%)	
III	15 (14.3%)	2 (22.2%)	
IV	4 (3.8%)	0 (0.0%)	

^*^Mean ± standard deviation.

^†^Use Fisher exact test.

^‡^Available data only.

The 5-year OS rate of the 153 melanoma patients was 57.8%. When stratified by stress-bearing (n = 105) and non-stress-bearing (n = 9) melanomas, the 5-year OS rates of the melanoma patients were 55.8% and 65.6%, respectively. Kaplan-Meier curves revealed no significant OS, DMFS, and RFS differences between melanomas developed in stress-bearing and non-stress-bearing areas (*P* = 0.6, 0.64, and 0.97, respectively, log rank test) (Fig. 1c and Supplementary Fig. S2). There was also no significant difference in OS between the rear/front of the foot group and the arch group (*P* = 0.945). The results of Cox univariate and multivariate analyses of the OS, DMFS, and RFS rates are shown in Table 2 and Supplementary Tables S2,3 and cover 6 factors (age, gender, Breslow thickness, ulceration, lymph node metastasis, and tumor distribution). The positive lymph node, ulceration, and thickness factors were associated with OS, DMFS, and RFS in the univariate analysis. In the multivariate analysis, the positive lymph node and thickness factors remained the most crucial prognostic factors for predicting OS and DMFS. Furthermore, gender and lymph node status were associated with RFS in the multivariate analysis.

**Table 2:** Univariate and multivariate analyses results of risk factors associated with overall survival.

Variables	Univariate HR (95% CI)	Univariate *P*-value	Multivariate HR (95% CI)	Multivariate *P*-value
Age, y*	1.02 (1.00, 1.04)	0.056	1.02 (1.00, 1.05)	0.07
Male gender	1.54 (0.90, 2.61)	0.113	1.03 (0.54, 1.96)	0.93
Positive lymph node	6.82 (3.72, 12.53)	<0.0001	7.55 (3.34, 17.05)	<0.0001
Ulceration	1.85 (1.06, 3.22)	0.03	0.64 (0.30, 1.36)	0.25
Thickness, mm*	1.11 (1.05, 1.17)	0.0001	1.08 (1.01, 1.16)	0.034
Stress-bearing area	1.38 (0.41, 4.59)	0.603	1.25 (0.37, 4.25)	0.72

CI, confidence intervals; HR, hazard ratio.

^*^Continuous variables.

## Discussion

Acral melanomas are the most commonly occurring subtype of melanoma in Asians and have many unique characteristics compared with the cutaneous melanomas seen elsewhere in terms of presentation and prognosis^12, 13^. Acral melanomas may present a greater diagnostic challenge because the lesions often are presented and diagnosed late, adversely affecting outcomes^12^. The present study constitutes one of the largest studies of acral melanomas to date, including data on 153 foot melanomas included in a multi-center database for Taiwan. The mean age of the melanoma patients in question was 68 years, which was similar to the mean ages of patients in previous Japanese and Chinese studies but older than the mean age of patients in a Korean study of acral melanomas^1, 5–7^. The mean Breslow thickness in this study was 4.1 mm, which was similar to the mean thickness reported for the aforementioned Chinese data but much thicker than the mean thickness values of other previous studies regarding cutaneous melanomas, suggesting that acral melanomas had a more advanced thickness^1^. There was a slight female predominance among the melanoma patients in this study (male-to-female ratio: 1: 1.28), consistent with previous reports^7, 14^. Compared with those of Korean and Chinese patients, the acral melanomas of the Taiwanese patients in this study had a lower rate of ulceration (32.7% vs. 42.3% and 47.9%) and a lower proportion of melanomas thicker than 4 mm (25.5% vs. 32.9% and 40.8%), findings which might explain the higher 5-year survival rate of the patients in this study (57.8% vs. 49.3% and 53.3%)^1, 5^. Compared to those previously seen in Caucasians, meanwhile, the acral melanomas of the Taiwanese patients in this study were usually diagnosed at a late stage and resulted in a lower survival rate^14^.

Melanomas on volar sites are of interest because they arise on skin that receives little or no sun exposure, unlike cutaneous melanomas on other anatomic sites^15–17^. A recent study showed that acral melanomas have distinct genetic features, with a lower frequency of ultraviolet-induced characteristics^18^. Due to their anatomical locations, it has been hypothesized that trauma might be a predisposing factor for acral melanomas^5^. Supporting this hypothesis, the subsite distribution of acral melanomas is noticeable^19^. Mechanical stresses such as plantar pressure and shear stress are higher on the front and rear areas than on other areas of the sole^6, 20^. This may be an important feature in determining where skin breakdown develops more frequently. One more crucial factor may be that volar skin is hypopigmented even in black skin^16^. This hypopigmentation could be a considerable cause of melanomas in volar skin, given that melanin molecules function as a blocker of the free radicals generated locally by inflammation^16^.

In a previous study of 47 cases, plantar melanomas were noted to be more common on the weight-bearing areas of the soles in a Caucasian population^7^. The relevance of mechanical stress to melanomas was also later reported in Koreans patients, but that study only conducted anatomic mapping of the distribution of melanomas and did not include data on the RFS and DMFS rates^5^. Minagawa *et al*. established an analysis method to quantitatively evaluate the association between mechanical stress points and melanomas^6^. However, their sample size was small and they did not analyze clinicopathological factors other than thickness^6^. Furthermore, a survival analysis was not included in their study^14^. Using a measure of goodness of fit, we found that stress-bearing areas, especially the rear and front of the foot, were significantly more conducive to the development of melanomas than non-stress-bearing areas^6^. As in previous reports, there were no significant differences between the genders regarding the distinct sites on the sole on which melanomas were found, as well as no differences between left and right soles in our study^7^. In addition, the prognostic indexes including ulceration, thickness, mitosis, positive lymph node, and stage did not differ significantly between the stress- and non-stress-bearing portions^5^. Although the acral melanomas tended to develop on stress-bearing-areas, their distribution pattern was not associated with death, metastasis, or recurrence^5^. The focus of this study, as well as previous studies, was on investigating the link between mechanical stress and acral melanomas on the soles, hence whether the research findings can be applied to acral melanomas found on the hand is still a matter of uncertainty^6, 7, 12, 21^. Further research involving the collection of enough data on palmar melanoma cases is required. The etiology of acral melanomas remains unclear, but several risk factors have been identified, including penetrative injury, heavy exposure to agricultural chemicals, and ethnicity^5, 15^. Future molecular studies are needed to determine the mechanism through which mechanical stress is associated with higher incidences of melanomas.

## Methods

### Patients and tissues

This retrospective cohort report was approved by the Research Ethics Committee of National Taiwan University Hospital (NTUH-REC No.: 201508050RIND) and it was performed according to the principles of the Declaration of Helsinki. Five hundred and seventeen consecutive pathology reports regarding melanomas diagnosed between January 1, 2000, and December 31, 2015, at National Taiwan University Hospital and Kaohsiung Chang Gung Memorial Hospital were obtained using a computer-assisted search. All the cases were evaluated by at least 2 pathology faculty members at the time of diagnosis. Pathology reports from the study period were evaluated, and biopsies from specimens that were not adequate to check the lesion precisely were omitted (n = 63). Cases involving the local recurrence or metastasis of melanomas (n = 130) were likewise excluded from the study, as were melanomas not classified as acral melanomas (n = 77)^6^. In addition, cases for which clinical photographs and clinical data (medical history) were lacking were excluded (n = 74) from the study. Since the relevant clinical photographs were not available, these cases could not be appropriately categorized into groups for further analysis. Cases involving lesions located on the hands were excluded (n = 20) due to the relatively small sample size for this type of lesion^5, 6^. After excluding the cases above, 153 acral melanomas remained (Supplementary Fig. S3). Of these, 13 cases were from Kaohsiung Chang Gung Memorial Hospital. All the patients who were enrolled in the study provided written informed consent and had standard-of-care treatment^22^. Data were collected on Breslow’s thickness, ulceration, mitosis, and lymph node status^8, 23^. The mitotic rate was categorized as follows: <1 mitosis/mm^2^, 1–6 mitosis/mm^2^, and >6 mitosis/mm^2^ ^5^. Clinical data describing patient demographics, the distribution and size of lesions, the American Joint Committee on Cancer stage, the clinical course, and follow-up through November 30, 2016, were assessed after being collected from the medical records and the Cancer Registry of the Medical Information Management Office of Kaohsiung Chang Gung Memorial Hospital and National Taiwan University Hospital. Overall survival was defined as the time from diagnosis to death or the last follow-up^8^. Distant metastasis-free survival was regarded as the length of time after therapy during which no distant metastases were noted. Recurrence-free survival was defined as the length of time after therapy during which no local recurrence or regional, or distant metastasis was noted.

### Statistical analysis

Descriptive statistics were used to summarize the data. Associations between the distribution of lesions and clinicopathological factors were evaluated with the Fisher exact test, Chi-square test, or goodness of fit test, when indicated. Survival probabilities were assayed using the Kaplan-Meier method and were evaluated by log-rank tests^22^. The influence of each factor on survival was calculated using univariate analysis by the Cox proportional hazard models. In light of the fact that thickness, ulceration, and lymph node metastasis are features considered in staging, the stage factor was not examined in the Cox regression models. Because of missing values in many cases, mitosis was also excluded from the Cox regression models. All tests were two-sided. *P* values of less than 0.05 were regarded as statistically significant. Analysis were performed using SAS 9.4 (Cary, North Carolina, USA).

## Electronic supplementary material

Supplementary information
