# Supplementary material for: A clinicopathological analysis of 153 acral melanomas and the relevance of mechanical stress
Source: Sci Rep. 2017 Jul 17;7:5564. doi: 10.1038/s41598-017-05809-9 (PMC5514126; doi:10.1038/s41598-017-05809-9)
Supplement: Supplementary file 1 — Supplementary information [file 41598_2017_5809_MOESM1_ESM.doc]

**A clinicopathological analysis of 153 acral melanomas and the relevance of mechanical stress**

Yi-Shuan Sheen, MD^1, 2^; Yi-Hua Liao, MD, PhD^2^; Ming-Hsien Lin, MD^3, 4^; Jau-Shiuh Chen, MD^2^; Jau-Yu Liau, MD^1^; Yu-Ju Tseng^5^; Chih-Hung Lee^5^; Yih-Leong Chang^1^, MD ^*¶1^; Chia-Yu Chu, MD, PhD ^*¶2^

| **Supplementary Table S1. Patient clinicopathological data** | |
| --- | --- |
| **Parameters** | **No. (%)** |
| Gender |  |
| Male | 67 (43.8%) |
| Female | 86 (56.2%) |
| Age at diagnosis, y (mean) | 68 |
| 20-29 | 1 (1.0%) |
| 30-39 | 3 (2.0%) |
| 40-49 | 14 (9.2%) |
| 50-59 | 23 (15.0%) |
| 60-69 | 39 (25.5%) |
| 70-79 | 39 (25.5%) |
| ≥80 | 34 (22.2%) |
| Right/left | 74 (48.4%)/79 (51.6%) |
| Thickness, mm (mean) |  |
| In situ | 21 (13.7%) |
| ≤1 | 23 (15.0%) |
| 1.01-2.0 | 37 (24.2%) |
| 2.01-4.0 | 33 (21.6%) |
| >4 | 39 (25.5%) |
| Ulceration |  |
| Present | 50 (32.7%) |
| Absent | 103 (67.3%) |
| Mitosis (n=77)^*^ |  |
| <1 | 19 (24.7%) |
| 1-6 | 46 (59.7%) |
| >6 | 12 (15.6%) |
| Lymph node metastasis |  |
| Present | 27 (17.6%) |
| Absent | 126 (82.4%) |
| **(Continued)** |  |
| **Supplementary Table S1. Patient clinicopathological data (Continued)** | |
| **Parameters** | **No. (%)** |
| AJCC stage |  |
| 0 | 20 (13.1%) |
| I | 45 (29.4%) |
| II | 60 (39.2%) |
| III | 22 (14.4%) |
| IV | 6 (3.9%) |
| Site of lesion |  |
| Sole | 127 (83.0%) |
| Front of foot | 37 (24.2%) |
| Midfoot | 16 (10.5%) |
| Rear of foot | 52 (34.0%) |
| Arch | 9 (5.9%) |
| Borders | 13 (8.5%) |
| Toes | 26 (17.0%) |
| Plantar aspect | 15 (9.8%) |
| Subungual and periungual | 11 (7.2%) |

^*^Available data only.

| **Supplementary Table S2. Univariate and multivariate analyses results of risk factors associated with distant metastasis-free survival** | | | | |
| --- | --- | --- | --- | --- |
| **Variables** | **Univariate HR**  **(95% CI)** | **Univariate**  ***P*-value** | **Multivariate HR (95% CI)** | **Multivariate**  ***P*-value** |
| Age, y* | 1.00 (0.98, 1.02) | 0.95 | 1.00 (0.98, 1.02) | 0.95 |
| Male gender | 1.32 (0.74, 2.36) | 0.36 | 1.53 (0.78, 3.01) | 0.22 |
| Positive lymph node | 11.52 (5.99, 22.15) | <0.0001 | 9.49 (3.98, 22.61) | <0.0001 |
| Ulceration | 2.46 (1.37, 4.44) | 0.0027 | 0.86 (0.39, 1.9) | 0.7 |
| Thickness, mm* | 1.13 (1.07, 1.19) | <0.0001 | 1.10 (1.03, 1.18) | 0.0043 |
| Stress-bearing area | 0.78 (0.28, 2.20) | 0.64 | 1.34 (0.46, 3.93) | 0.59 |

CI, confidence intervals; HR, hazard ratio.

^*^Continuous variables

| **Supplementary Table S3. Univariate and multivariate analyses results of risk factors associated with recurrence-free survival** | | | | |
| --- | --- | --- | --- | --- |
| **Variables** | **Univariate HR**  **(95% CI)** | **Univariate *P*-value** | **Multivariate HR**  **(95% CI)** | **Multivariate**  ***P*-value** |
| Age, y* | 1.01 (0.99, 1.03) | 0.29 | 1.01 (0.99, 1.04) | 0.24 |
| Male gender | 1.64 (0.98, 2.74) | 0.059 | 2.24 (1.22, 4.13) | 0.0095 |
| Positive lymph node | 10.85 (6.02, 19.56) | <0.0001 | 13.38 (5.91, 30.31) | <0.0001 |
| Ulceration | 2.64 (1.57, 4.44) | 0.0002 | 0.97 (0.49, 1.94) | 0.93 |
| Thickness, mm* | 1.11 (1.06, 1.16) | <0.0001 | 1.06 (1, 1.13) | 0.053 |
| Stress-bearing area | 1.02 (0.37, 2.85) | 0.97 | 1.22 (0.41, 3.6) | 0.72 |

CI, confidence intervals; HR, hazard ratio.

^*^Continuous variables


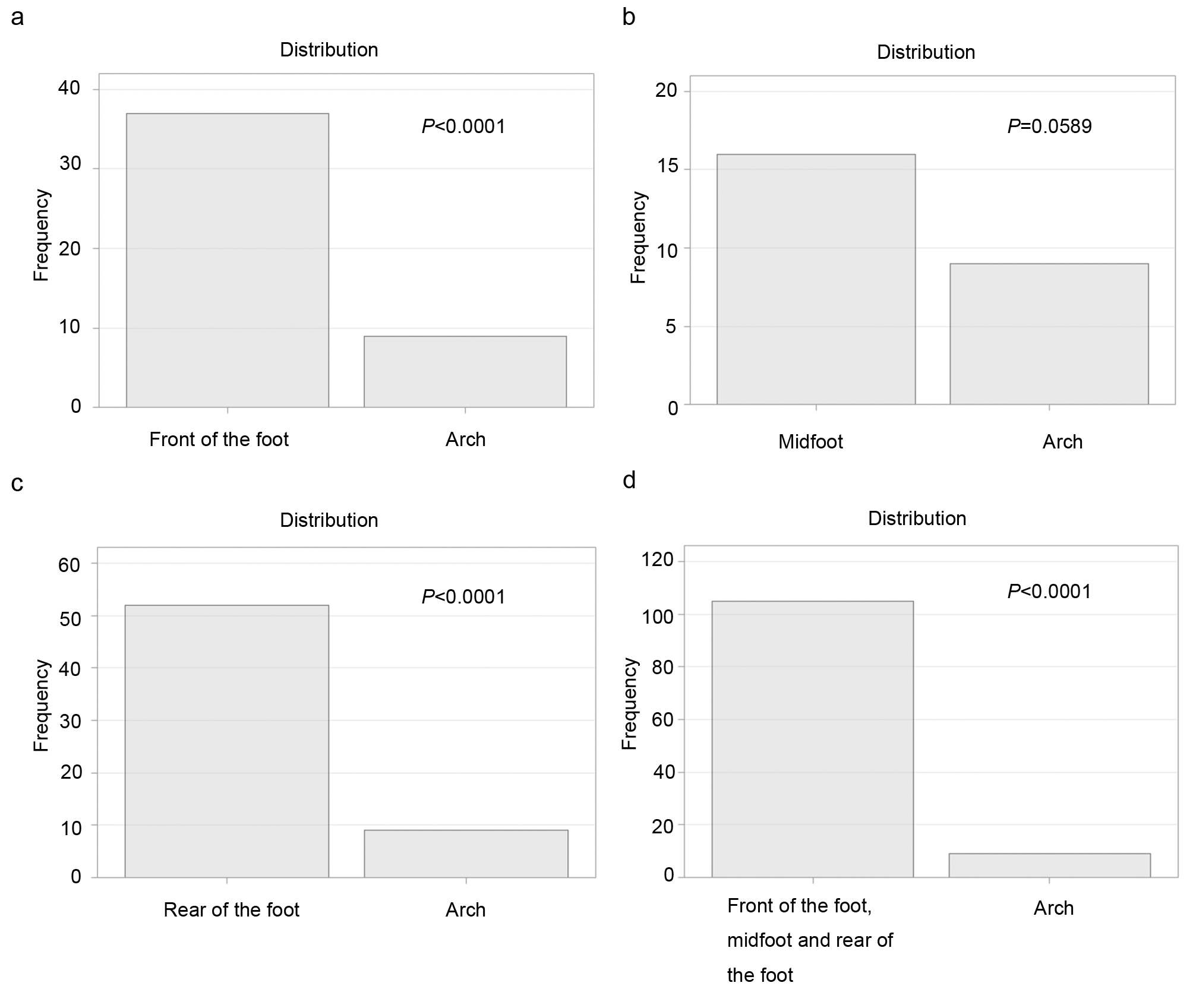


**Supplementary Figure S1**. Distribution of 114 primary acral melanomas in the plantar area. Goodness of fit was used to compare the observed sample distribution with the expected probability distribution. Compared to the arch, the rear of the foot and front of the foot were significantly more conductive to the generation of melanomas (*P*<0.0001 & *P*<0.0001, respectively), but the midfoot was not significantly more conductive to the generation of melanomas (*P*=0.059). Stress-bearing areas were significantly more conducive to the growth of melanomas than non-stress-bearing areas (*P*<0.0001).


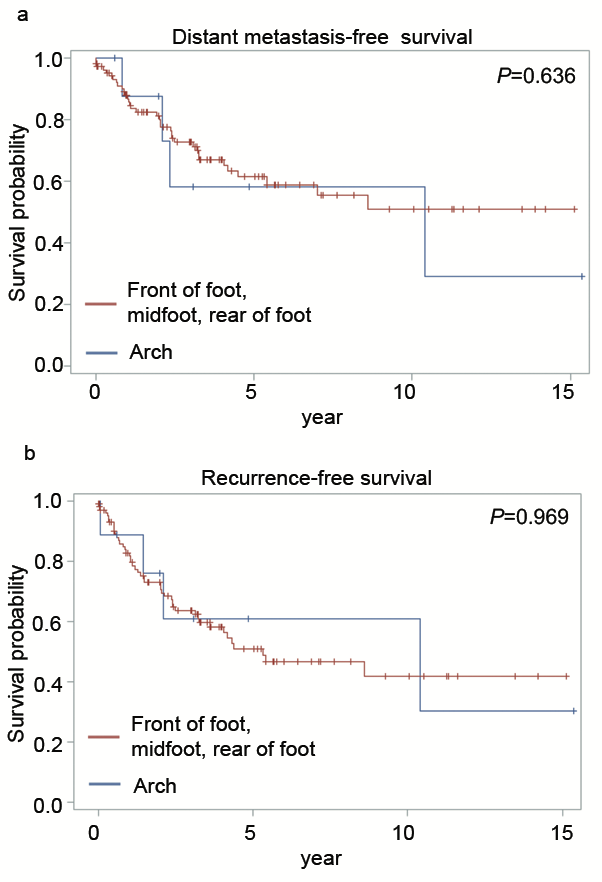


**Supplementary Figure S2**. Kaplan-Meier curves of survival in 114 primary acral melanomas. No distant metastasis-free and recurrence-free survival differences were found between melanomas developed in stress-bearing and non-stress-bearing areas (*P*=0.636 and 0.969, respectively, log-rank test).


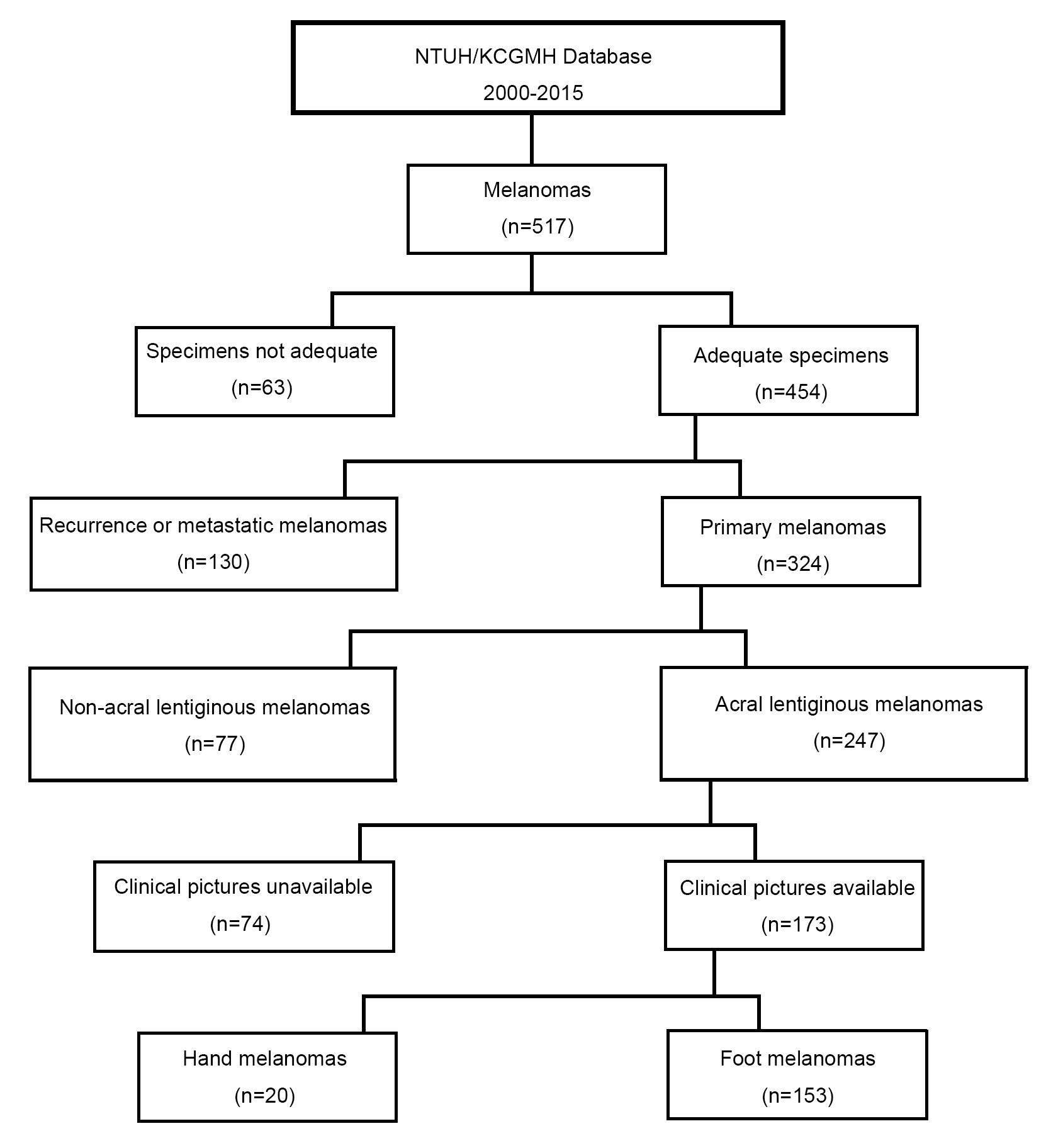


**Supplementary Figure S3. Study flow diagram.** Five hundred and seventeen consecutive pathology reports regarding melanomas were obtained. Pathology reports from the study period were evaluated, and biopsies from specimens that were not adequate to check the lesion precisely were omitted (n=63). Cases involving the local recurrence or metastasis of melanomas (n=130) were likewise excluded from the study, as were melanomas not classified as acral melanomas (n=77). In addition, cases without adequate clinical pictures and medical history were excluded (n=74). Cases involving lesions located on the hands were excluded (n=20). After excluding the cases above, 153 acral melanomas remained. NTUH: National Taiwan University Hospital; KCGMH: Kaohsiung Chang Gung Memorial Hospital.
